# Performance comparison of different machine learning models to predict the risk of postcraniotomy nausea and vomiting: a retrospective study

**DOI:** 10.3389/fmed.2026.1901505

**Published:** 2026-09-07

**Authors:** Lingyi Wu, Hongying Pan, Yihong Xu, Yushu Sun

**Affiliations:** Department of Nursing, Sir Run Run Shaw Hospital, Zhejiang University School of Medicine, Hangzhou, Zhejiang, China

**Keywords:** craniotomy, machine learning, PONV, postoperative nausea and vomiting, risk prediction model

## Abstract

**Background:**

Machine learning (ML) models were constructed and validated to predict the risk of postoperative nausea and vomiting (PONV) after craniotomy. We compared different algorithms to provide a reliable tool for clinical decision-making.

**Methods:**

Data from patients who underwent craniotomy at a tertiary hospital in Zhejiang Province between January 1, 2021 and December 31, 2022 were retrospectively collected as the training set. LASSO (Least Absolute Shrinkage and Selection Operator) regression with 10-fold cross-validation was used to screen for key predictors, and four prediction models were established: Logistic Regression (LR), Random Forest (RF), eXtreme Gradient Boosting (XGBoost), and Light Gradient Boosting Machine (LightGBM). Patients from September 1, 2023 to December 22, 2023 served as the temporal validation set. Evaluation of model performance was based on accuracy, precision, specificity, recall, F1-score, Brier score, and the area under the receiver operating characteristic curve (AUC). Temporal validation was performed to assess the generalization of the models and decision curve analysis (DCA) was applied to evaluate clinical utility.

**Results:**

A total of 768 eligible samples were included, and 14 core predictors were screened by LASSO regression. Of the four constructed risk prediction models, temporal validation based on ML algorithms confirmed that LightGBM achieved optimal comprehensive performance with the highest accuracy, recall, F1-score, and AUC, along with lowest Brier score, indicating that this model had significant advantages in discriminative, calibration, and generalization capabilities. The LR model demonstrated stable performance with high specificity but low recall, indicating a certain risk of missed diagnosis, while the performance of the RF model decreased significantly from the training to validation set, suggesting obvious overfitting and limited clinical application value. The XGBoost model achieved moderate performance, but had a relatively higher Brier score, suggesting moderate prediction error. The DCA results showed that LightGBM offered greater and more stable net benefits across a wider range of clinically relevant threshold probabilities, further supporting its clinical utility.

**Conclusion:**

LightGBM shows the best overall performance for risk stratification of PONV after craniotomy, and SHAP visualization endows the model with favorable clinical interpretability to facilitate individualized risk prediction.

## Introduction

Postoperative nausea and vomiting (PONV) was defined as subjective nausea, vomiting or retching after surgery, symptoms typically develops within 24 h and may persist for up to 48 h or longer in a small number of cases (1, 2). As one of the most common distressing complications following surgical procedures reported by patients, PONV can lead to a series of adverse consequences, including electrolyte disturbance, airway injury, venous hypertension, wound dehiscence, and hematoma at the surgical site (3, 4). The incidence of PONV among patients undergoing neurosurgical craniotomy ranges from 39 to 80% (1, 5–7). During the projectile phase of vomiting, the resulting high intra-abdominal and intrathoracic pressure (>100 mmHg) is directly transmitted to the intracranial cavity, which can induce increased intracranial pressure, elevate the risk of intracranial hemorrhage, and even lead to life-threatening complications, such as cerebral herniation (8–10). These sequelae not only prolong the length of hospital stay but also increase the medical burden. Therefore, accurate identification of patients at high risk of PONV and implementation of individualized preventive strategies are crucial to improve clinical outcomes.

Machine learning (ML) is a hot field in artificial intelligence research that can learn and summarize rules from big data, and construct prediction models by processing large clinical datasets combined with multiple indicators to evaluate outcome risks (11, 12). Many recent studies have employed various ML algorithms to construct and compare models in order to determine the optimal model. As compared to traditional algorithms, ML algorithms can more efficiently process high-dimensional and large-scale data and capture nonlinear relationships among data, thereby more accurately predicting the occurrence of diseases (13). However, most of the currently used risk scores for PONV are universal tools, which have not been validated on a large scale in neurosurgical patients and lack specificity. In China, research on PONV risk prediction models after neurosurgery is relatively recent and there have been no reports on PONV risk prediction models based on ML algorithms or comparisons of the performance of multiple models (12). Therefore, prediction models for postcraniotomy nausea and vomiting were constructed based on four algorithms: logistic regression (LR), random forest (RF), extreme gradient boosting (XGBoost), and light gradient-boosting machine (LightGBM). The selected four algorithms comprehensively cover the spectrum of clinical prediction modeling approaches: from the simple and interpretable (LR) through bagging ensembles (RF) to the state-of-the-art gradient boosting methods (XGBoost, LightGBM). The aim of this study was to identify the optimal prediction model, recognize craniotomy patients at potential risk of PONV as early as possible, formulate individualized preventive measures, and provide a reference for standardized perioperative management.

## Materials and methods

### Study population and data collection

This single-center retrospective study employed a temporal validation protocol. The training set and validation set were obtained from the same institution but enrolled during different time periods to eliminate sample overlap and ensure independence. All data were collected and managed in strict accordance with the inclusion and exclusion criteria, which guaranteed the reliability of model training and validation.

Patients who underwent craniotomy at a tertiary hospital in Zhejiang Province, China were retrospectively enrolled. Patients enrolled between January 1, 2021 and December 31, 2022 were assigned to the training set, whereas those admitted from September 1, 2023 to December 31, 2023 constituted the temporal validation set for temporal validation. The inclusion criteria were as follows: (1) age ≥ 18 years and (2) hospitalization after craniotomy under general anesthesia. The exclusion criteria were as follows: (1) severe diseases, such as heart failure, liver or kidney failure, or end-stage disease, (2) critically ill patients who underwent tracheostomy, (3) patients who underwent a second surgery for reasons, such as postoperative intracranial hemorrhage, progression of severe cerebral edema, or hydrocephalus, (4) patients who experienced postoperative changes in their condition with nausea and vomiting accompanied by a decreased level of consciousness, changes to pupil size, or changes to muscle strength, (5) patients classified as level 5 according to the American Society of Anesthesiologists’ Physical Status Classification (ASA classification) of 2020, (6) incomplete clinical data, and (7) vulnerable populations, including critically ill patients and pregnant women. The study protocol was approved by the hospital’s ethics committee (Hospital Ethics Review 2023 Research No. 0433).

### Outcome definition

The primary outcome was postoperative nausea and vomiting (PONV) occurring within the first 24 h postoperatively. PONV was defined as nausea, retching, or vomiting occurring in the postoperative period. Nausea was defined as a patient-reported subjective sensation of urge to vomit. Vomiting was defined as the observed expulsion of gastric contents or documented retching in the medical records. Retching refers to the dry heaving movement without expulsion of gastric contents, and is considered clinically equivalent to vomiting. (3, 14, 15).

Observation period was the first 24 h after surgery, with data extracted from standardized nursing records and anesthesia charts.

Classification was binary: patients with any documented nausea or vomiting during the observation period were classified as PONV-positive (coded as 1), while those without were classified as PONV-negative (coded as 0).

### Study material

#### Stage 1: clinical pre-selection

Based on a literature review and expert consultation, predictors associated with the risk of postcraniotomy PONV were identified. Data were retrospectively collected by the investigators from the hospital’s electronic medical record system.

The 36 variables mainly included sex, age, body mass index, smoking history, history of motion sickness or PONV, antecedent history, ASA class, preoperative electrolyte indicators (preoperative serum potassium, sodium, chloride, calcium, and blood glucose), operation type, surgical site, surgical time, intraoperative position, drainage tube, hemorrhage, intraoperative transfusion, anesthesia time, anesthetic drug, intraoperative remifentanil dosage, intraoperative sufentanil dosage, intraoperative laboratory examinations (intraoperative blood gas pH, potassium, sodium, chloride, calcium, blood glucose, and lactate), postoperative pain severity, postoperative opioids, postoperative antiemetic, postoperative steroid, neostigmine, and pneumocephalus.

Model prediction time: Given that the earliest PONV onset in the training cohort was 65 min postoperatively. The prediction timepoint of our model is set at 1 h after anesthesia emergence, aiming to predict subsequent PONV events from 1 h to 24 h post-craniotomy. All postoperative predictors including postoperative steroids, opioids, antiemetics, pain scores and pneumocephalus CT results were assessed before the standardized first PONV screening at 1 h post-anesthesia.

#### Stage 2: LASSO regularization

To reduce dimensionality and prevent overfitting, we applied LASSO (Least Absolute Shrinkage and Selection Operator) with 10-fold cross-validation on the training set. The optimal penalty parameter *λ* was selected at the minimum cross-validated deviance (λ_min). Features with non-zero coefficients at λ_min were retained.

### Sample size calculation

The sample size was estimated using the “events per variable” method. According to expert consensus (12), the incidence of PONV following neurosurgery ranges from 47 to 70%. In this study, 14 predictive variables were finally included after screening by LASSO regression. Assuming a follow-up loss rate of 10%, the estimated total sample size was calculated as follows: (10 × 14 ÷ 0.47) / (1–10%) ≈ 331cases. The training and validation sets included 665 and 103 patients, respectively, which met the sample size requirements of the present study.

### Data preparation and preprocessing

The collected data were entered into an Excel file and double-checked to ensure accuracy, followed by standardized data cleaning. Data cleaning was performed following a predefined protocol (16).

#### Missing data handling

Missing values were addressed using Multiple Imputation by Chained Equations (MICE) with 5 imputations and 20 iterations. Continuous variables were imputed using predictive mean matching (PMM), binary variables using logistic regression, and multi-category variables using multinomial regression.

#### Sensitivity analysis

Due to the lack of routine intraoperative blood gas tests, the missing rate of relevant indicators exceeded 20%. To verify the reliability of missing data processing methods, we conducted sensitivity analysis. Complete case analysis was performed to assess the robustness of the MICE imputation results.

#### Feature scaling

Z-score standardization (center and scale) was applied only to the LR model, as tree-based models (RF, XGBoost, and LightGBM) are insensitive to feature scales. The validation set was scaled using training set parameters.

#### Categorical encoding

One-hot encoding was applied using model.matrix(), with consistent dummy variables ensured across training and validation sets via intersection of common columns.

#### Outlier management

Outliers were detected using the IQR method (1.5 × IQR). No data points were excluded based solely on outlier status.

#### Class imbalance treatment

ROSE oversampling was applied when the event rate fell below 20% or exceeded 80%. The training set event rate was 27.22%, and the oversampling was unapplied.

### Model construction and evaluation

#### Model selection

The selection of the four machine learning algorithms—Logistic Regression (LR), Random Forest (RF), XGBoost, and LightGBM—was based on a systematic framework covering four key dimensions: (1) linear vs. non-linear modeling (LR vs. tree-based models); (2) interpretability vs. predictive performance; (3) bagging vs. boosting ensemble paradigms; and (4) comparison of state-of-the-art boosting variants.

LR was included as the clinical gold standard due to its direct interpretability as odds ratios and regulatory acceptance. RF was selected for its bagging ensemble mechanism, built-in feature importance, and out-of-bag error estimation. XGBoost was chosen for its proven performance with built-in L1/L2 regularization and native missing value handling. LightGBM was included for its computational efficiency and superior performance in our preliminary experiments (AUC = 0.862).

#### Data splitting

The dataset was divided into a training set and an independent temporal validation set. The training set comprised the original development cohort (*n* = 665) and was used exclusively for model development, including data preprocessing, feature engineering, variable selection, hyperparameter tuning, and model training. The validation set consisted of an independent temporal cohort (*n* = 103) and was completely isolated from all training procedures.

#### Cross-validation

To optimize model performance and prevent overfitting, 5-fold cross-validation was employed for hyperparameter tuning exclusively on the training set. LASSO variable selection employed 10-fold cross-validation. The optimal hyperparameters were selected based on the highest cross-validated AUC.

#### Hyperparameter tuning

LR underwent grid search for alpha ({0, 0.5, 1}) and lambda (10^−4^ to 10^0^), with the optimal parameters selected as default values. RF was tuned for mtry ({2, 4, 6, 8, 10}). LightGBM was tuned for num_leaves ({5, 10, 15, 31}) and learning_rate ({0.01, 0.05, 0.1}). XGBoost was tuned for eta ({0.01, 0.05, 0.1, 0.2}) and max_depth ({2, 3, 4, 6}).

#### Overfitting prevention

Multiple strategies were implemented: (1) LASSO regularization for feature selection; (2) early stopping with 15-round patience for LightGBM and XGBoost; (3) L1/L2 regularization for all models; (4) subsampling (60%) and column sampling (60%) for tree-based models; (5) independent external validation; and (6) Bootstrap validation with 50 resamples for optimism assessment.

### Statistical analysis

Data analysis and modeling were performed using R Studio software (version 4.3.2), while baseline data were analyzed using IBM SPSS (version 27.0). Continuous data with a normal distribution were compared with the independent samples *t*-test and are reported as the mean ± standard deviation. Continuous data with a non-normal distribution were compared with nonparametric tests and are presented as the median (interquartile range). A probability (*p*) value < 0.05 was considered statistically significant. The optimal predictive variables were screened using LASSO regression combined with 10-fold cross-validation. With lambda.min (the *λ* value corresponding to the minimum cross-validation deviation) as the screening criterion, variables with non-zero coefficients were retained, and four predictive models were constructed based on these variables. The performance of each model was comprehensively evaluated using accuracy, precision, specificity, recall, F1-score, Brier score, and AUC value. Model performance was compared using Delong’s test for paired ROC curves. All performance metrics are reported with 95% confidence intervals calculated using bootstrap resampling with 1,000 iterations. The random seed was fixed at 123 for all analyses to ensure reproducibility. SHAP (Shapley Additive Explanations) values were calculated to quantify the contribution of each predictive factor to the model output, visually presenting the interpretability of the risk prediction model.

## Results

### Basic characteristics

**Figure 1:**
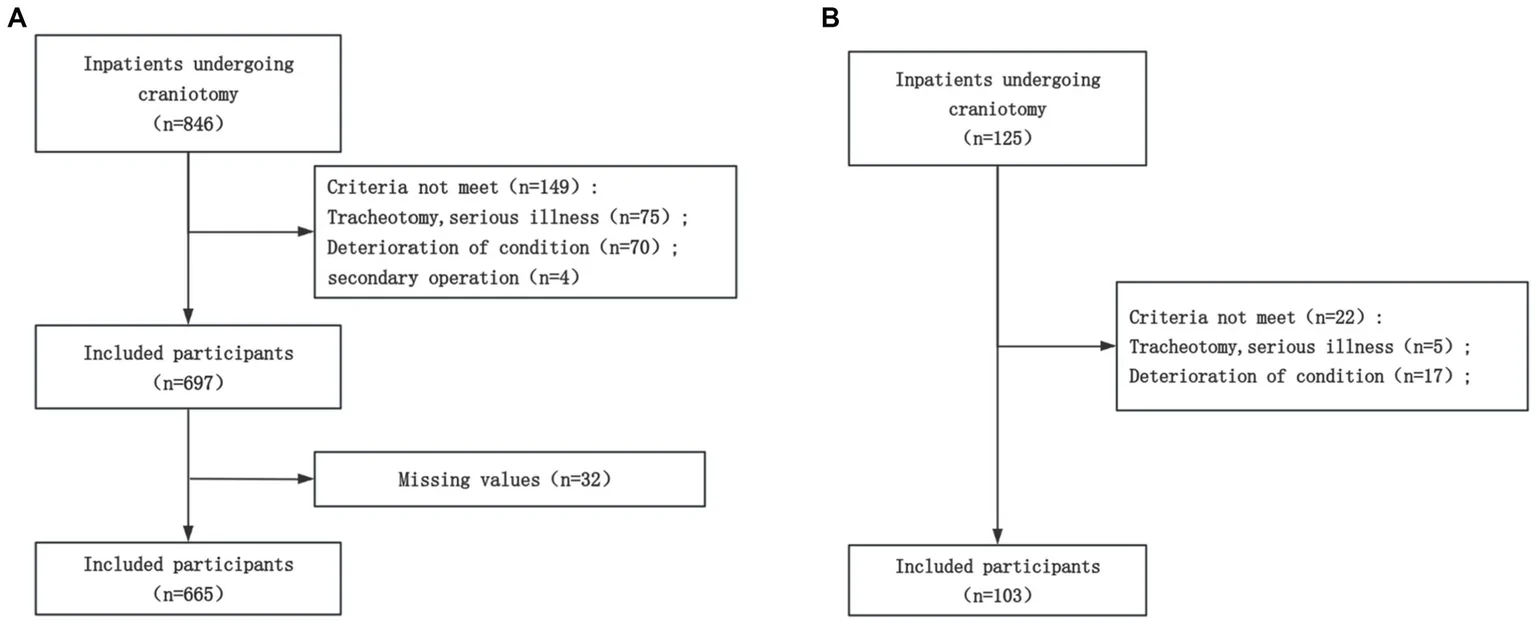
Modeling group subject screening **(A)**. Validation group subject screening **(B)**.

The study cohort included 768 patients (374 males and 394 females; age range, 18–93 years). PONV occurred in 222 patients, yielding an incidence rate of 28.9%. The dataset was temporally partitioned, with 665 patients assigned to the training set and 103 to the validation set. There were no statistically significant differences in baseline characteristics between the training and validation sets (*p* > 0.05) (Table 1). The patient selection flowchart (Figure 1) illustrates the patient enrollment and exclusion process in this study.

**Table 1:** Comparison of clinical characteristics between the training and validation sets.

Variable	Modeling group (*n* = 665)	Validation group (*n* = 103)	Statistic	*p*
Sex, n (%)			χ2 = 0.032	0.859
Male	323 (48.6)	51 (49.5)		
Female	342 (51.4)	52 (50.5)		
Age, median (IQR), y	57 (49, 66)	61 (53, 67.5)	Z = −2.008	0.045
BMI, median (IQR), kg/m^2^	23.7 (21.6, 25.8)	23.4 (22.2, 25.7)	Z = −0.041	0.967
Smoke, n (%)			χ2 = 0.630	0.427
Yes	118 (17.7)	15 (14.6)		
No	547 (82.3)	88 (85.4)		
History of motion sickness or PONV, n (%)			χ2 = 0.348	0.555
Yes	65 (9.8)	12 (11.7)		
No	600 (90.2)	91 (88.3)		
Antecedent history, n (%)			χ2 = 6.003	0.306
Upper gastrointestinal tract disease	17 (2.6)	1 (1.0)		
Hypertension	238 (35.8)	39 (37.9)		
Diabetes	19 (2.9)	6 (5.8)		
Clinical depression	11 (1.7)	1 (1.0)		
Other diseases	124 (18.7)	24 (23.3)		
Unrecorded	255 (38.4)	32 (31.1)		
ASA class, n (%)			χ2 = 3.534	0.316
I	2 (0.3)	0 (0.0)		
II	508 (76.4)	86 (83.5)		
III	146 (22.0)	17 (16.5)		
IV	9 (1.4)	0 (0)		
V	0 (0)	0 (0)		
Preoperative potassium, median (IQR), mmol/L	3.94 (3.72, 4.19)	3.94 (3.66, 4.11)	Z = −1.309	0.191
Preoperative sodium, median (IQR), mmol/L	140 (139, 142)	141 (139, 141.5)	Z = −0.468	0.639
Preoperative blood chloride, median (IQR), mmol/L	105 (103, 107)	105 (103, 106)	Z = −1.139	0.255
Preoperative calcium, mean ± SD, mmol/L	2.31 ± 0.11	2.29 ± 0.1	t = 2.229	0.027
Preoperative blood glucose, median (IQR), mmol/L	5.39 (4.92, 6.42)	5.25 (4.85, 6.07)	Z = −1.908	0.056
Surgical site, n (%)			χ2 = 6.043	0.014
Craniotomy on the curtain	450 (67.7)	57 (55.3)		
Craniotomy under the curtain	215 (32.3)	46 (44.7)		
Operation type, n (%)			χ2 = 2.519	0.641
Microvascular decompression	113 (17.0)	22 (21.4)		
Salpingectomy (medicine)	321 (48.3)	48 (46.6)		
Hematoma removal	188 (28.3)	26 (25.2)		
Cerebrovascular surgery	32 (4.8)	4 (3.9)		
Other surgeries	10 (1.5)	3 (2.9)		
Surgical time, n (%), min			χ2 = 0.008	0.928
<100	73 (11.0)	11 (10.7)		
≥100	592 (89.0)	92 (89.3)		
Drainage tube, n (%)			χ2 = 3.571	0.312
Subdural drain	189 (28.4)	38 (36.9)		
Subcutaneous drain	133 (20.0)	21 (20.4)		
Lumbar large pool drain or lateral extraventricular drain	321 (48.3)	41 (39.8)		
Drainless	22 (3.3)	3 (2.9)		
Intraoperative position, n(%)			χ2 = 3.314	0.191
Supine	422 (63.5)	58 (56.3)		
Lateral	30 (4.5)	3 (2.9)		
Prone	213 (32.0)	42 (40.8)		
Hemorrhage, median(IQR), mL	100 (20, 200)	50 (20, 100)	Z = −1.560	0.119
Intraoperative blood transfusion, n (%)			χ2 = 0.087	0.768
Yes	37 (5.6)	5 (4.9)		
No	628 (94.4)	98 (95.1)		
Anesthesia time, n (%), min			χ2 = 0.055	0.815
<200	162 (24.4)	24 (23.3)		
≥200	503 (75.6)	79(76.7)		
Anesthetic drug, n (%)			χ2 = 119.205	< 0.001
Ketorolac (sevoflurane)	12 (1.8)	4 (3.9)		
Sevoflurane liquid	625 (94.0)	62 (60.2)		
Other anesthetics	28 (4.2)	37 (35.9)		
Intraoperative remifentanil dosage, median (IQR), mg	2 (2, 2)	2 (2, 2)	Z = −1.548	0.122
Intraoperative sufentanil dosage, median (IQR), ug	50 (50, 50)	50 (50, 50)	Z = −1.969	0.049
Intraoperative blood gas pH	7.42 (7.4, 7.44)	7.42 (7.39, 7.44)	Z = −1.443	0.149
Intraoperative potassium, median (IQR), mmol/L	3.65 (3.5, 3.8)	3.59 (3.4, 3.8)	Z = −2.582	0.010
Intraoperative sodium, median (IQR), mmol/L	142.63 (142, 144)	141.45 (140, 143)	Z = −4.243	p < 0.001
Intraoperative blood chloride, median (IQR), mmol/L	108.93 (107, 110)	107.27 (106, 109.5)	Z = −4.162	< 0.001
Intraoperative calcium, median (IQR), mmol/L	1.04 (1.01, 1.08)	1 (0.98, 1.06)	Z = −4.380	< 0.001
Intraoperative blood glucose, median (IQR), mmol/L	6.3 (5.6, 6.5)	6.09 (5.5, 6.3)	Z = −2.715	0.007
Intraoperative blood gas lactate, median (IQR), mmol/L	1.93 (1.3, 2)	1.65 (1.1, 1.85)	Z = −3.580	< 0.001
Postoperative antiemetic, n (%)			χ2 = 1.053	0.305
Yes	574 (86.3)	85 (82.5)		
No	91 (13.7)	18 (17.5)		
Neostigmine, n (%)			χ2 = 2.606*	0.106
Yes	8 (1.2)	4(3.9)		
No	657 (98.8)	99 (96.1)		
Postoperative steroid, n (%)			χ2 = 0.258	0.612
Yes	305 (45.9)	50 (48.5)		
No	360 (54.1)	53 (51.5)		
Postoperative pain, n (%)			χ2 = 2.601	0.272
Mild	628 (94.7)	101 (98.1)		
Moderate	28 (4.2)	1 (1.0)		
Severe	7 (1.1)	1 (1.0)		
Postoperative opioids, n (%)			χ2 = 6.161	0.013
Yes	181 (27.2)	16 (15.7)		
No	484 (72.8)	86 (84.3)		
Pneumocephalus, n (%)			χ2 = 1.128	0.7288
Yes	198 (29.8)	36 (35.0)		
No	467 (70.2)	67 (65.0)		

*Continuity correction.

BMI, body mass index; ASA, American Society of Anesthesiologists; PONV, postoperative nausea and vomiting; IQR, interquartile range.

### Variable screening

**Figure 2:**
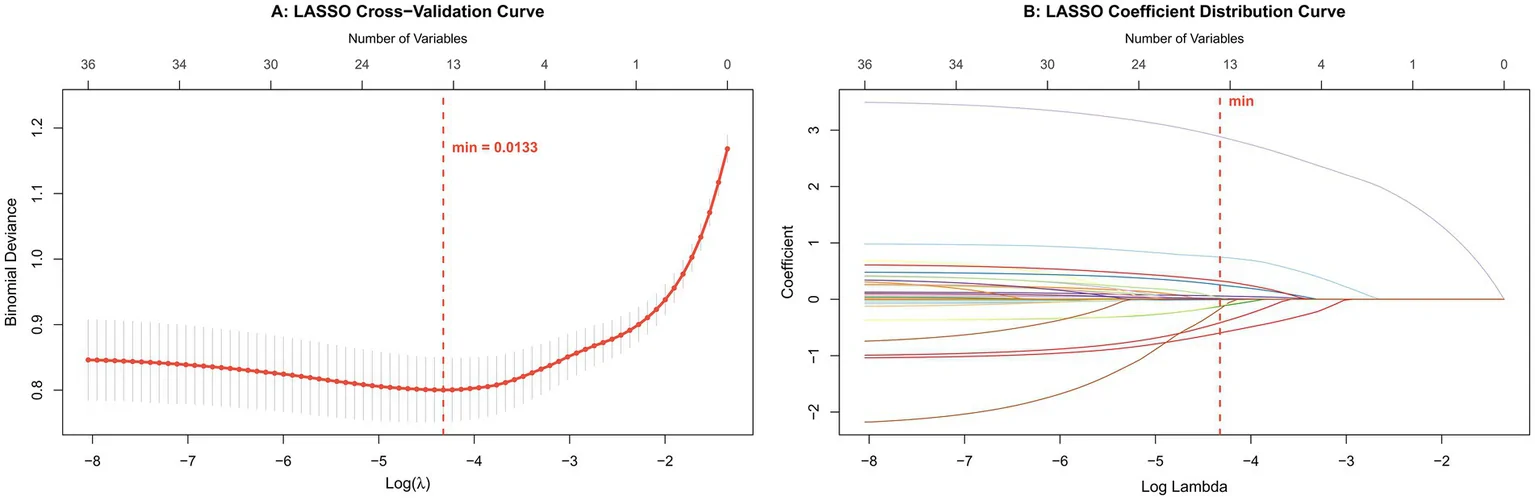
LASSO regression model and coefficient distribution plot of risk factors for PONV after craniotomy. **(A)** LASSO cross-validation curve. The red dashed line indicates lambda.min (λ = 0.0133), the optimal regularization parameter corresponding to the minimum binomial deviance. **(B)** LASSO coefficient distribution curve. The red dashed line corresponds to lambda.min, showing the coefficient trajectories of predictive variables across different λ values. Variables with non-zero coefficients at lambda.min were retained for subsequent model construction.

The 36 variables initially included in this study were screened to identify optimal predictive factors using LASSO regression combined with 10-fold cross-validation. With lambda.min (*λ* = 0.0133) as the optimal criterion, 14 predictive variables with non-zero coefficients were finally identified: sex, age, smoking history, history of motion sickness or PONV, preoperative sodium, chloride, operation type, intraoperative calcium, sodium, anesthesia time, postoperative steroid, postoperative opioids, pneumocephalus and postoperative antiemetic.(Figure 2).

Following the missing data sensitivity analysis, the complete case analysis demonstrated consistent findings with the MICE imputation approach. All absolute AUC differences across the four models were below the clinically acceptable threshold of 0.02 (range: 0.003 to 0.011), indicating that the missing data handling strategy did not substantially impact model performance. These results confirm the robustness of the MICE multiple imputation results.

LASSO Stability Assessment: Bootstrap stability assessment (100 resamples) showed a mean selection frequency of 59.8% (SD: 22.4%), 7 variables were selected in more than 80% of bootstrap samples, indicating robust selection for these core predictors.

### Construction and validation of a risk prediction model for post-craniotomy PONV

In this study, PONV risk prediction models were constructed and validated based on four ML algorithms. The performance of LR, RF, XGBoost, and LightGBM models was comprehensively evaluated based on seven indicators: accuracy, precision, specificity, recall, F1 score, Brier score, and AUC, which systematically reflected the overall predictive ability of the models.

**Figure 3:**
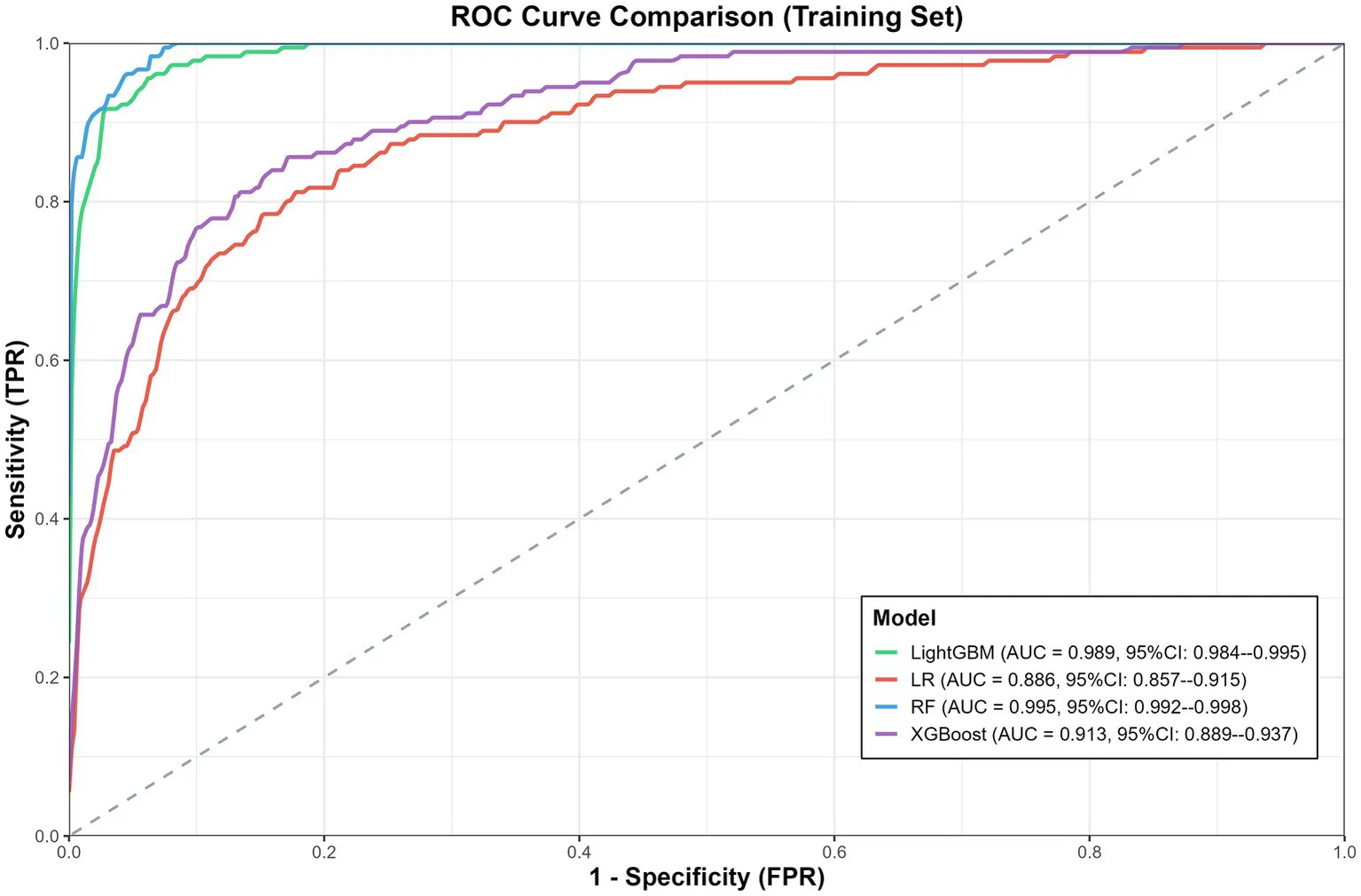
ROC curves of the four ML models with the training set. LR, logistic regression; RF, random forest; XGBoost, extreme gradient boosting; LightGBM, light gradient-boosting machine; AUC, area under the ROC curve.

**Figure 4:**
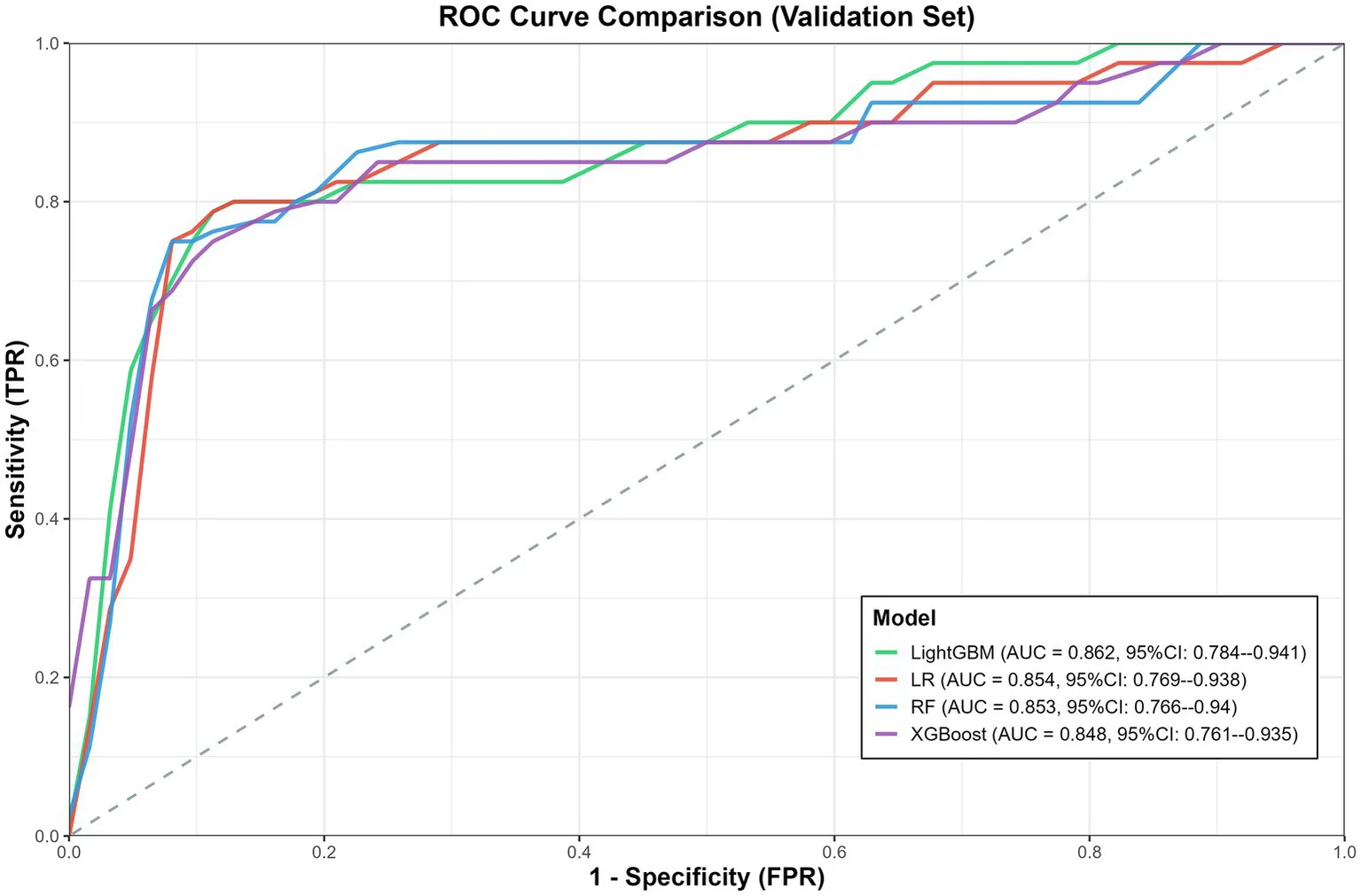
ROC curves of the four ML models with the validation set. LR, logistic regression; RF, random forest; XGBoost, extreme gradient boosting; LightGBM, light gradient-boosting machine; AUC, area under the ROC curve.

According to the comprehensive evaluation results, the LightGBM model had the optimal comprehensive performance with the highest accuracy, recall, F1-score, and AUC, along with the lowest Brier score, indicating significant advantages in discriminative ability, calibration ability, and generalization. The LR model showed stable performance with high specificity but low recall, indicating a certain risk of missed diagnosis. The performance of the RF model decreased significantly from the training set to the validation set, suggesting obvious overfitting and limited clinical application value. The XGBoost model achieved moderate performance, but had a relatively higher Brier score (0.158), suggesting moderate prediction error. The temporal validation results directly reflect the generalization ability of the models with an independent dataset, which provides key evidence of clinical practical value. The specific indicators of each model are shown in Table 2 and the ROC curves of the training and validation sets are shown in Figure 3 and Figure 4, respectively.

**Table 2:** Specific performance indicators of the four prediction models with the training and validation sets.

Model	Accuracy	Precision	Specificity	Recall	F1 score	Brier score	AUC	Dataset
LR	0.848	0.756	0.921	0.652	0.7	0.112	0.886	Training
RF	0.952	0.987	0.996	0.834	0.904	0.046	0.995	Training
LightGBM	0.95	0.93	0.975	0.884	0.906	0.047	0.989	Training
XGBoost	0.86	0.824	0.95	0.619	0.707	0.112	0.913	Training
LR1	0.775	0.84	0.935	0.525	0.646	0.171	0.854	Validation
RF1	0.755	0.857	0.952	0.45	0.59	0.169	0.853	Validation
LightGBM1	0.824	0.893	0.952	0.625	0.735	0.149	0.862	Validation
XGBoost1	0.814	0.889	0.952	0.6	0.716	0.158	0.848	Validation

All Metrics with 95%CI							
Model	Accuracy (95% CI)	Precision (95% CI)	Specificity (95% CI)	Recall (95% CI)	F1 score (95% CI)	AUC (95% CI)	Dataset
LR1	0.775 (0.696–0.853)	0.84 (0.692–0.963)	0.935 (0.873–0.985)	0.525 (0.366–0.678)	0.646 (0.5–0.767)	0.854 (0.758–0.928)	Validation
RF1	0.755 (0.676–0.824)	0.857 (0.688–1)	0.952 (0.897–1)	0.45 (0.292–0.595)	0.59 (0.431–0.721)	0.853 (0.755–0.929)	Validation
LightGBM1	0.824 (0.745–0.882)	0.893 (0.771–1)	0.952 (0.893–1)	0.625 (0.472–0.771)	0.735 (0.6–0.835)	0.862 (0.778–0.93)	Validation
XGBoost1	0.814 (0.735–0.882)	0.889 (0.758–1)	0.952 (0.897–1)	0.6 (0.433–0.75)	0.716 (0.571–0.825)	0.848 (0.751–0.928)	Validation

### Clinical utility analysis of the postcraniotomy PONV prediction model

#### Decision curve analysis

**Figure 5:**
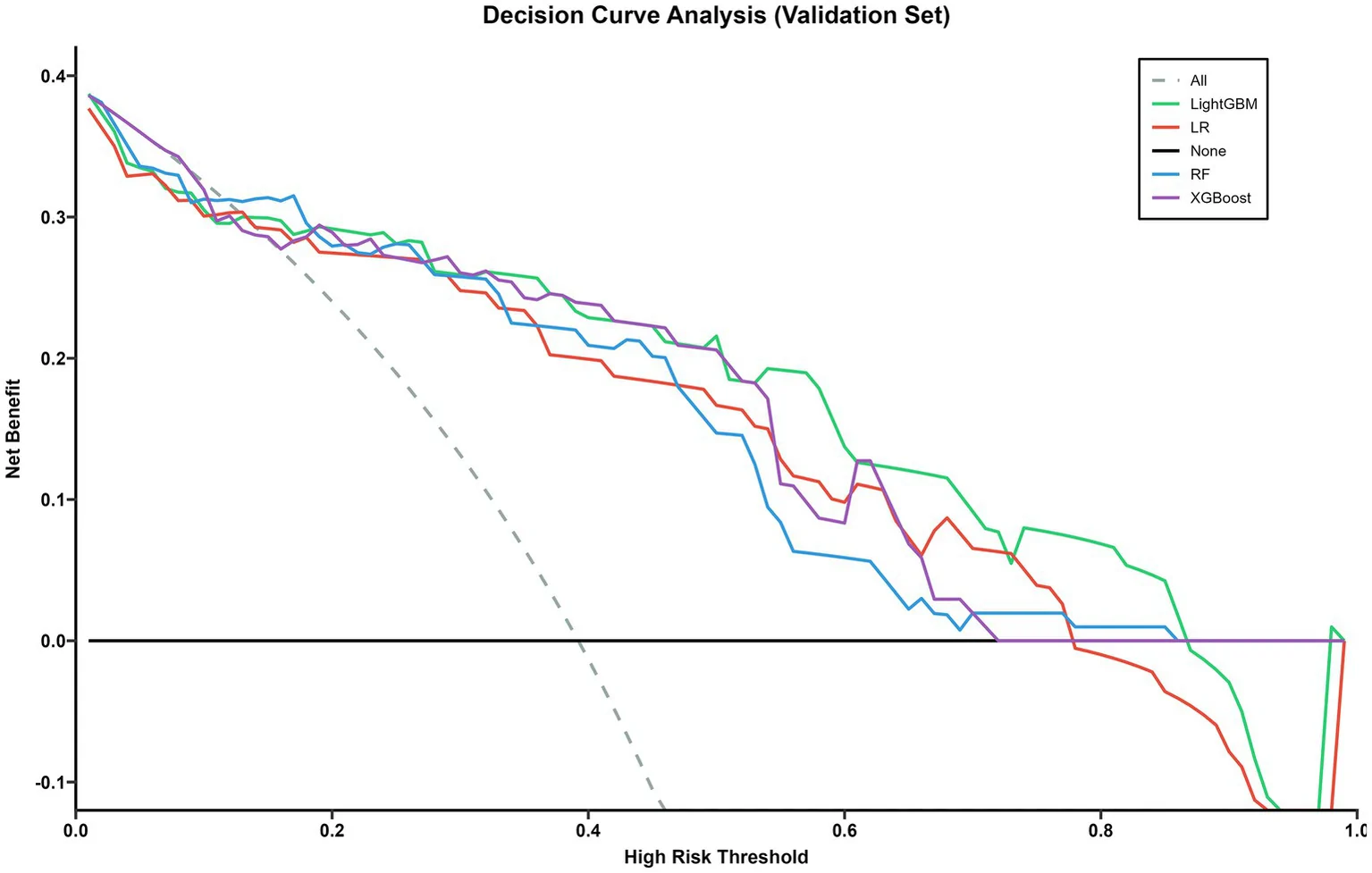
DCA comparing the clinical net benefits of the four models to predict postoperative PONV with the validation set. The *x*-axis indicates the threshold probability and the *y*-axis represents the net benefit. The black solid line represents the assumption that all patients are at high risk and the black dashed line represents the assumption that no patients are at high risk. LR, logistic regression; RF, random forest; XGBoost, extreme gradient boosting; LightGBM, light gradient-boosting machine.

Decision curve analysis (DCA) showed that LightGBM and LR models achieved positive net benefits across a wide range of threshold probabilities. Of these, the LightGBM model exhibited a consistently higher net benefit than the LR model at moderate-to-high threshold probabilities (0.20–0.85), suggesting superior clinical value for individualized risk assessment as the most clinically useful prediction model in this study (Figure 5).

#### Delong test

Delong’s test revealed no statistically significant differences between LightGBM and any other model (all *p* > 0.05; Table 3), indicating that while LightGBM achieved numerically higher AUC(0.862), the differences were not statistically significant.

**Table 3:** Delong test.

Comparison	Model 1	Model 2	AUC1	AUC2	AUC_Diff	Z_Statistic	P_value	Significant
LR vs RF	LR	RF	0.854	0.853	0	0.019	0.9852	No (P ≥ 0.05)
LR vs LightGBM	LR	LightGBM	0.854	0.862	−0.008	−0.349	0.7273	No (P ≥ 0.05)
LR vs XGBoost	LR	XGBoost	0.854	0.848	0.005	0.237	0.8124	No (P ≥ 0.05)
RF vs LightGBM	RF	LightGBM	0.853	0.862	−0.009	−0.414	0.6787	No (P ≥ 0.05)
RF vs XGBoost	RF	XGBoost	0.853	0.848	0.005	0.302	0.763	No (P ≥ 0.05)
LightGBM vs XGBoost	LightGBM	XGBoost	0.862	0.848	0.014	0.724	0.4693	No (P ≥ 0.05)

#### Calibration analysis

**Figure 6:**
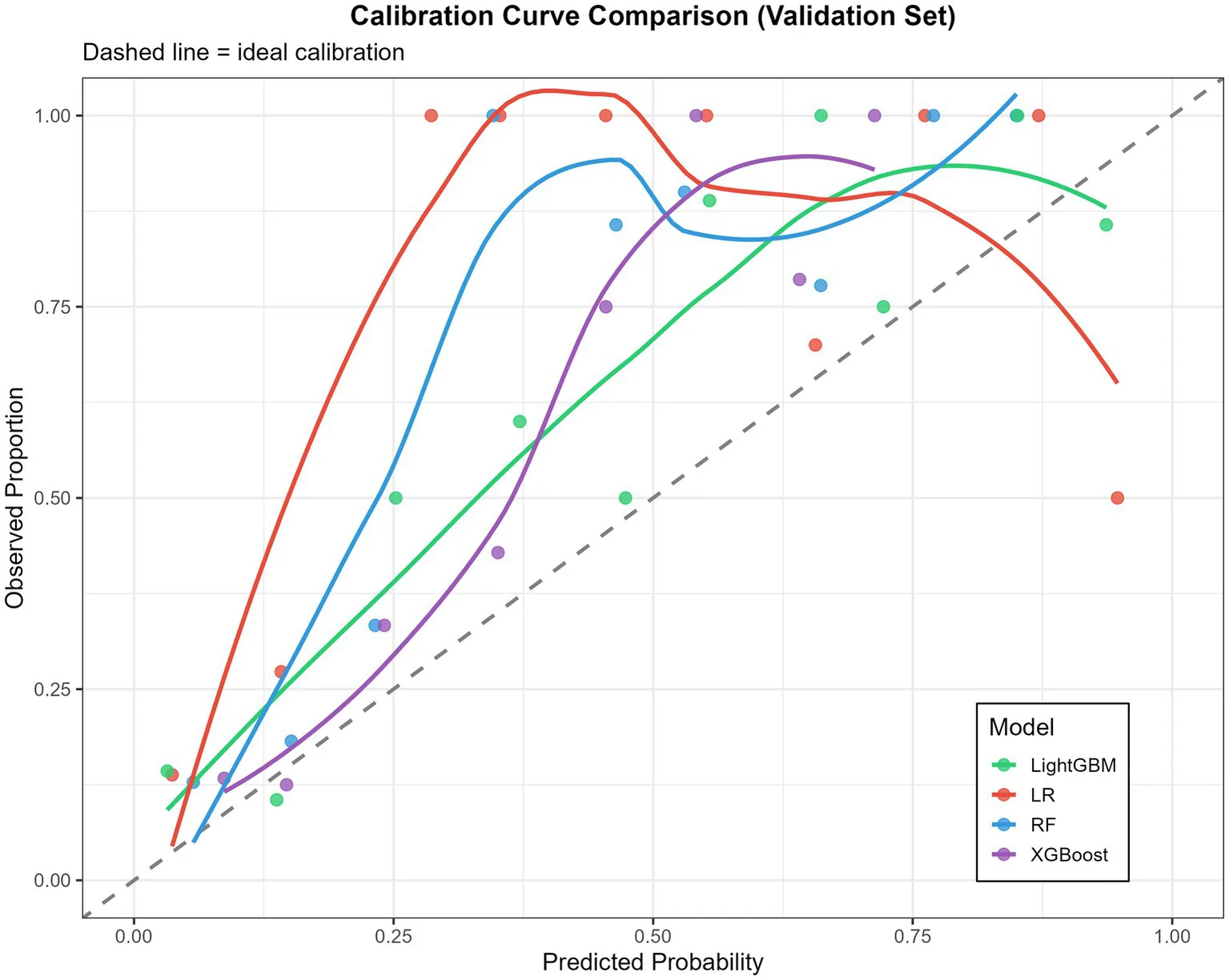
Calibration curves comparison of the four ML models. The dashed diagonal line represents ideal calibration (intercept = 0, slope = 1), where predicted probabilities perfectly match observed event rates. Points closer to the diagonal line indicate better calibration performance. Among the four models, LightGBM demonstrated the closest agreement with the ideal calibration line, followed by LR, XGBoost, and RF, suggesting that LightGBM provided the most accurate probability estimates for PONV risk prediction.

Calibration was assessed using calibration curves, calibration intercept, calibration slope, and the Hosmer-Lemeshow (HL) test (Table 4, Figure 6).

**Table 4:** Calibration analysis summary.

Model	Dataset	Calibration_Intercept	Calibration_Slope	HL_Pvalue	HL_Statistic	N_Bins
LR	Validation	1.059	0.81	0	47.432	10
RF	Validation	1.275	1.251	0.004	22.752	9
LightGBM	Validation	0.672	0.794	0.001	27.18	10
XGBoost	Validation	1.103	1.568	0.083	13.97	8

Based on the calibration analysis, LightGBM demonstrated the best overall calibration performance in the validation set. It achieved the calibration intercept closest to 0 (0.672) and the calibration slope closest to 1 (0.794) among all models, indicating the smallest systematic bias in probability estimation. Although XGBoost had the highest Hosmer-Lemeshow *p*-value (0.083), suggesting better HL test performance, its calibration slope (1.568) showed the most extreme prediction probabilities, indicating over-dispersion. Considering the comprehensive calibration assessment, LightGBM was selected as the optimal model.

### Visualization of the postoperative PONV risk prediction model for craniotomy patients

We have now incorporated SHAP (SHapley Additive exPlanations) analysis for the best-performing LightGBM model to enhance interpretability and clinical transparency. Specifically, we calculated SHA*p* values for all features in the validation set and generated:a SHAP importance ranking table.

**Figure 7:**
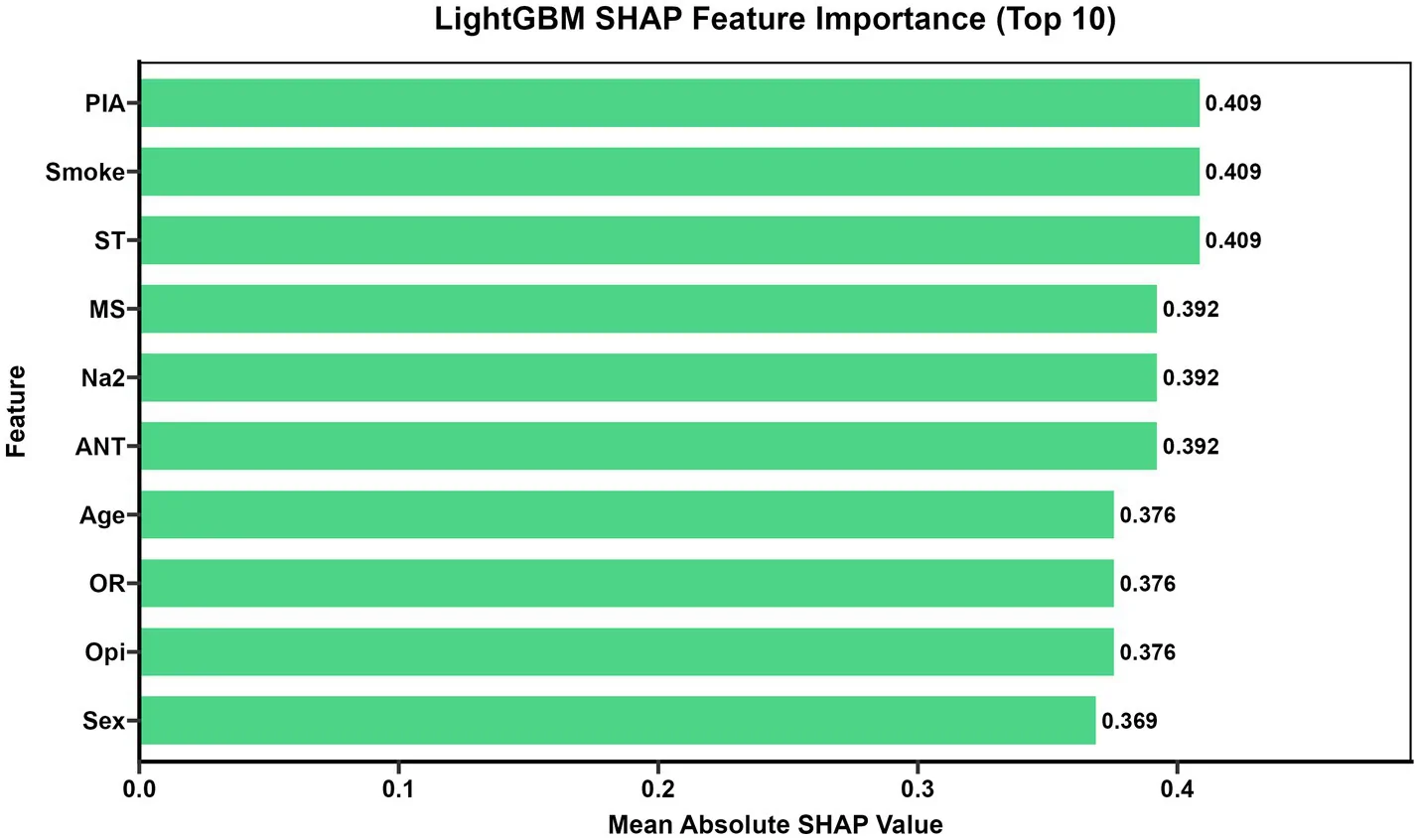
SHAP feature importance plot (top 10) for PONV risk factors based on the LightGBM model. The top 10 feature variables in order of importance were pneumocephalus, smoking history, postoperative steroid, history of motion sickness or PONV, intraoperative sodium, postoperative antiemetic, age, operation type, postoperative opioids, sex (Abbreviations: PIA, Postoperative Intracranial Air, ST, postoperative steroid, MS, history of motion sickness or PONV, Na2, intraoperative sodium; ANT, postoperative antiemetic; OR, operation type; Opi, postoperative opioids.).

The LightGBM model was employed to rank the importance of each variable associated with PONV risk factors. The top 10 feature variables in order of importance were pneumocephalus, smoking history, postoperative steroid, history of motion sickness or PONV, intraoperative sodium, postoperative antiemetic, age, operation type, postoperative opioids, sex (Figure 7).

The LightGBM model demonstrated superior comprehensive performance for both statistical metrics and clinical net benefit, as the optimal technical choice for this study. In contrast, the LR model, owing to its irreplaceable interpretability, serves as a bridge between clinical translation and physician comprehension. Risk-stratified prophylactic intervention based on predicted probabilities can prevent unnecessary costs and potential medication-related adverse effects (17).

### Comparison with existing clinical prediction tools

Previous studies (18) has demonstrated that machine learning-based prediction models for postoperative nausea and vomiting (PONV) in neurosurgical patients exhibit superior overall predictive performance compared with the Apfel score. In this study, we initially planned to compare the predictive performance of our craniotomy-specific PONV risk prediction model with the Apfel score. However, upon retrospective data collection, we found that documentation of Apfel score components was incomplete in over 54.3% of cases, precluding a valid comparative analysis. This finding suggests that the Apfel score is not widely implemented in our institutional clinical practice. Future prospective studies with larger sample sizes are warranted to compare the predictive performance of the two approaches (Table 5).

**Table 5:** Univariate analysis results in the development cohort.

Variable	PONV group (*n* = 181)	Non-PONV group (*n* = 484)	Statistic	*P* values
Sex, n(%)			χ2 = 34.952	< 0.001
Male	54 (29.8)	269 (55.6)		
Female	127 (70.2)	215 (44.4)		
Age, median (IQR), y	56 (48, 64)	57 (49, 67)	Z = -2.091	0.037
BMI, median (IQR), kg/m^2^	23.6 (21.4, 25.4)	23.9 (21.67, 25.9)	Z = -1.287	0.198
Smoke, n(%)			χ2 = 8.949	0.003
Yes	19 (10.5)	99 (20.5)		
No	162 (89.5)	385 (79.5)		
History of motion sickness or PONV, n(%)			χ2 = 1.895	0.169
Yes	13(7.2)	52(10.7)		
No	168(92.8)	432(89.3%)		
Antecedent history, n(%)			χ2 = 5.021	0.413
Upper gastrointestinal tract disease	2 (1.1)	15 (3.1)		
Hypertension	57 (31.5)	181 (37.4)		
Diabetes	5 (2.8)	14 (2.9)		
Clinical depression	3 (1.7)	8 (1.7)		
Other diseases	39 (21.5)	85 (17.6)		
unrecorded	75 (41.4)	181 (37.4)		
ASA class, n(%)			χ2 = 8.139	0.043
I	0 (0)	2 (0.4)		
II	151 (83.4)	357 (73.8)		
III	27 (14.9)	119 (24.6)		
IV	3 (1.7)	6 (1.2)		
V	0 (0)	0 (0)		
Preoperative potassium, median (IQR), mmol/L	3.93 (3.76, 4.22)	3.94 (3.7, 4.18)	Z = -0.687	0.492
Preoperative sodium, median (IQR), mmol/L	141 (139, 142)	140 (139, 142)	Z = -2.027	0.043
Preoperative blood chloride, median (IQR), mmol/L	106 (104, 107)	105 (103, 107)	Z = -3.011	0.003
Preoperative calcium, mean ± SD, mmol/L	2.32 ± 0.1	2.31 ± 0.12	t = −0.665	0.506
Preoperative blood glucose, median (IQR), mmol/L	5.27 (4.8, 5.95)	5.44 (4.97, 6.63)	Z = -2.377	0.017
Surgical site, n(%)			χ2 = 11.851	< 0.001
Craniotomy on the curtain	104 (57.5)	346 (71.5)		
Craniotomy under the curtain	77 (42.5)	138 (28.5)		
Type of surgery, n(%)			χ2 = 23.142	< 0.001
Microvascular decompression	35 (19.3)	78 (16.1)		
Salpingectomy (medicine)	96 (53)	226 (46.7)		
Hematoma removal	30 (16.6)	158 (32.6)		
Cerebrovascular surgery	16 (8.8)	16 (3.3)		
Other surgeries	4 (2.2)	6 (1.2)		
Surgical time,n(%), min			χ2 = 6.110	0.013
<100	11 (6.1)	62 (12.8)		
≥100	170 (93.9)	422 (87.2)		
Drainage tube, n(%)			χ2 = 24.927	< 0.001
Subdural drain	16 (8.8)	117 (24.2)		
Subcutaneous drain	88 (48.6)	233 (48.1)		
Lumbar large pool drain or lateral extraventricular drain	7 (3.9)	15 (3.1)		
Drainless	70 (38.7)	119 (24.6)		
Intraoperative position, n(%)			χ2 = 6.139	0.046
Supine position	109 (60.2)	313 (64.7)		
Lateral position	14 (7.7)	16 (3.3)		
prone position	58 (32.0)	155 (32.0)		
Hemorrhage, median(IQR), ml	100 (30, 200)	100 (20, 200)	Z = -0.709	0.478
Intraoperative blood transfusion, n(%)			χ2 = 0.166	0.684
Yes	9 (5)	28 (5.8)		
No	172 (95)	456 (94.2)		
Anesthesia time, n(%), min			χ2 = 4.197	0.041
<200	34 (18.8)	128 (26.4)		
≥200	147 (81.2)	356 (73.6)		
Anesthetic drug, n(%)			χ2 = 0.056	0.872
Ketorolac (sevoflurane)	170 (93.9)	455 (94.0)		
Sevoflurane liquid	8 (4.4)	20 (4.1)		
Other anesthetics	3 (1.7)	9 (1.9)		
Intraoperative remifentanil dosage, median(IQR), mg	2 (2, 2)	2 (2, 2)	Z = -1.486	0.137
Intraoperative sufentanil dosage, median(IQR), ug	50 (50, 50)	50 (50, 50)	Z = -2.275	0.023
Intraoperative blood gas pH	7.42 (7.4, 7.44)	7.42 (7.4, 7.44)	Z = -0.736	0.462
Intraoperative potassium, median(IQR), mmol/L	3.65 (3.5, 3.8)	3.65 (3.5, 3.8)	Z = -0.040	0.968
Intraoperative sodium, median(IQR), mmol/L	142.63 (142, 144)	142.63 (142, 143)	Z = -1.636	0.102
Intraoperative blood chloride, median(IQR), mmol/L	108.93 (108, 111)	108.93 (107, 110)	Z = -2.606	0.009
Intraoperative calcium, median(IQR), mmol/L	1.04 (1.01, 1.08)	1.04 (1.02, 1.08)	Z = -0.780	0.436
Intraoperative blood glucose, median(IQR), mmol/L	6.3 (5.5, 6.5)	6.3 (5.6, 6.5)	Z = -0.802	0.423
Intraoperative blood gas lactate, median(IQR), mmol/L	1.93 (1.6, 2.3)	1.93 (1.28, 1.93)	Z = -2.956	0.003
Postoperative antiemetic, n(%)			χ2 = 0.671	0.413
Yes	153 (84.5)	421 (87)		
No	28 (15.5)	63 (13)		
Neostigmine, n(%)			χ2 = 0*	1
Yes	2 (1.1)	6 (1.2)		
No	179 (98.9)	478 (98.8)		
Postoperative steroid, n(%)			χ2 = 1.505	0.220
Yes	76 (42.0)	229 (47.3)		
No	105 (58.0)	255 (52.7)		
Postoperative pain, n(%)			χ2 = 0.936	0.626
Mildly	171 (94.5)	459 (94.8)		
Moderately	7 (3.9)	21 (4.3)		
Severe	3 (1.7)	4 (0.8)		
Postoperative opioids, n(%)			χ2 = 2.293	0.130
Yes	57 (31.5)	124 (25.6)		
No	124 (68.5)	360 (74.4)		
Pneumocephalus, n(%)			χ2 = 227.197	< 0.001
Yes	133 (73.5)	65 (13.4)		
No	48 (26.5)	419 (86.6)		

* Continuity-corrected χ2 test. BMI, body mass index. ASA, American Society of Anesthesiologists. PONV, Postoperative Nausea and Vomiting. IQR, Interquartile Range.

## Discussion

### The risk prediction model shows favorable predictive performance for PONV

PONV is one of the most common complications within 24 h after craniotomy (7). Due to the unclear neurobiological mechanism, this “big minor problem” remains unresolved (19). Although aggressive prophylaxis and combination therapy have been used clinically to manage PONV, the incidence remains high (20). In the present study, the incidence of PONV was 27.2% (181 patients) in the training set and 39.8% (41 patients) in the validation set, which was generally consistent with the incidence reported both domestically and internationally (15, 21–23). Therefore, an effective and reliable risk prediction model is urgently needed for risk assessment and effective prevention of postoperative PONV following craniotomy.

The four risk prediction models constructed in this study all had an AUC > 0.845, indicating favorable ability to effectively distinguish between the PONV and non-PONV groups. The closer the AUC value is to 1, the better the predictive performance of the model. The Brier scores of the validation set were all less than 0.18 The closer the Brier score is to 0, the better the calibration ability and consistency of the model, verifying good reproducibility.

### Performance comparison of the four models

In recent years, ML has been widely applied in clinical practice. The LR model is sensitive to data with multicollinearity, tends to overfit, has limited classification accuracy, and faces difficulties in handling imbalanced data. The XGBoost model achieved high accuracy but the complexity makes results difficult to interpret. RF is a non-parametric ensemble method based on decision trees that shows excellent generalization for imbalanced data, with advantages of high speed and efficiency during data processing (24). Beyond performance metrics, the inherent algorithmic characteristics of each model directly influence clinical applicability. Although LR is sensitive to multicollinearity and limited in modeling nonlinear relationships, the high transparency and interpretability make it a gold standard for clinical communication and bedside teaching. The XGBoost and LightGBM models achieved outstanding accuracy in complex pattern recognition, but the “black-box” nature of the ensemble frameworks remains a major barrier to clinical acceptance.

In this study, prediction models were constructed using four ML algorithms: LR, RF, XGBoost, and LightGBM. The LightGBM model achieved the best overall performance, with the highest AUC (0.862) and optimal calibration (Brier score = 0.149) with the validation set, indicating an excellent balance between discrimination and predictive accuracy. The next best was the LR model. As a representative of classical statistical learning methods, it exhibited excellent robustness, with an outstanding AUC value of 0.854, which was close to that of the LightGBM model. However, its recall was relatively low (0.525), indicating a certain risk of missed diagnosis. This limitation should be emphasized in clinical application and result interpretation. In contrast, the RF model showed an obvious tendency of overfitting and insufficient generalization ability, suggesting insufficient optimization with the present dataset. Therefore, high caution should be exercised in clinical application of the RF model. Although LightGBM achieved the highest AUC (0.862) numerically, Delong’s test showed no statistically significant differences between LightGBM and other models (all *p* > 0.05). Nevertheless, LightGBM was selected as the preferred model due to its superior calibration performance and the highest net benefit across clinically relevant thresholds.

### Clinical value and stratified application strategy

#### Objective performance evaluation based on DCA

PONV presents a unique challenge to patients after craniotomy, not only due to a high incidence but also increased clinical risks (25). Although a substantial proportion of patients in our cohort had already received prophylactic dexamethasone and antiemetics, retrospective data collection revealed that 27.2% of craniotomy patients still developed PONV within the first 24 h, while prospectively collected data indicated an even higher incidence of 39.8%. These findings underscore the critical importance of identifying individuals at high risk for PONV and implementing effective perioperative prevention and management strategies.

Conventional discrimination metrics, such as the AUC, are insufficient to fully evaluate the clinical utility of a prediction model. To address this, DCA was employed to quantify the clinical net benefit of the models across a range of clinically reasonable threshold probabilities. The results demonstrated that the LightGBM model achieved the highest net benefit across the entire spectrum of threshold probabilities. This indicates that, when adopting “maximizing clinical benefit for patients” as the ultimate criterion, the LightGBM algorithm had the optimal combination of statistical performance and clinical utility in this study. Consequently, from both methodological and objective performance perspectives, LightGBM was identify as the optimal model. While the LightGBM model demonstrates promise as a decision support tool for personalized PONV prophylaxis, we acknowledge that several critical steps remain before clinical implementation. These include: (1) external validation in independent multicenter cohorts to confirm the generalizability of our findings; (2) prospective clinical utility studies, such as cluster-randomized trials, to demonstrate that model-guided prophylaxis improves patient outcomes; and (3) careful consideration of EHR integration, including workflow compatibility and user interface design. With these prerequisites satisfied, the LightGBM model may serve as a valuable decision support tool for personalized antiemetic prophylaxis in craniotomy patients (Table 6).

**Table 6:** LASSO vs. all feature compare.

Model	Dataset	AUC_LASSO	AUC_AllFeature	AUC_diff
LR	Validation	0.854	0.852	0.002
LightGBM	Validation	0.862	0.858	0.004
RF	Validation	0.853	0.862	−0.009
XGBoost	Validation	0.848	0.874	−0.026

### Clinical value of interpretability

#### Pathophysiological interpretation of key predictor

SHAP analysis identified pneumocephalus as the most influential predictor of PONV, a finding with important clinical and pathophysiological implications.

Under normal physiological conditions, the cranial cavity contains no free gas. During neurosurgical procedures performed in the sitting position, opening the dura mater allows direct entry of air into the intracranial space. Furthermore, gravitational sinking of brain tissue and excessive cerebrospinal fluid (CSF) loss reduce intracranial pressure, creating negative pressure that draws air into the cranial cavity—sometimes even resulting in tension pneumocephalus (26). CSF loss alters CSF pressure dynamics, which directly stimulates both the vomiting center and the chemoreceptor trigger zone, predisposing patients to postoperative nausea and vomiting (27). This mechanism is closely associated with surgical manipulation and postoperative low intracranial pressure following ventricular surgery. Previous studies have reported that patients undergoing microvascular decompression (MVD) surgery are at high risk for PONV, possibly due to intraoperative stimulation of the cerebellum and vestibular nerve, postoperative CSF hypotension, blood-stained CSF, and intracranial air accumulation (28).

Clinical Implications of This Finding:(1)Prevention strategies: Meticulous intraoperative technique to minimize dural defects, combined with postoperative head elevation and avoidance of hyperventilation to reduce intracranial air accumulation, may indirectly decrease PONV risk.(2)Risk assessment: When postoperative imaging reveals intracranial air, clinicians should maintain a high index of suspicion for PONV and initiate prophylactic antiemetic measures in a timely manner.(3)Intervention window: Early postoperative identification of pneumocephalus may provide a therapeutic window for PONV prevention, enabling precise perioperative management. Future studies should validate these findings in prospective multicenter cohorts and explore whether targeted interventions to prevent pneumocephalus may improve PONV outcomes (Table 7).

**Table 7:** Missing report detailed.

Variable	Train_Missing_Rate	Test_Missing_Rate
PH2	31.58	0
K2	31.28	0
Na2	30.98	0
Glu2	30.98	0
LA	30.98	0
Cl2	30.83	0
CA2	30.83	0
BMI	3.01	0
Na	0.3	0
Cl	0.3	0
Bleeding	0.3	0
K	0.15	0
CA	0.15	0
Sex	0	0
Age	0	0
Smoke	0	0
MH	0	0
MS	0	0
ASA	0	0
Glu	0	0
Route	0	0
OR	0	0
O-Time	0	0
BT	0	0
ST	0	0
Postion	0	0
AT	0	0
DT	0	0
Pain	0	0
Opi	0	0.97
Remifentanil	0	0
Sufentanil	0	0

#### Stratified clinical application framework

Temporal validation and the DCA results clearly revealed that the LightGBM model exhibited superior overall discrimination and population screening efficiency, whereas the LR model has natural advantages in individualized interpretation and doctor-patient communication. LR provides clear and stable odds ratios and a visualized nomogram, allowing physicians to perform rapid individualized assessment, bedside calculation, or teaching without electronic devices. This not only improves patients’ in-depth understanding of disease risk but also significantly enhances treatment adherence and satisfaction (29).

Accordingly, we propose a stratified application framework for the entire clinical decision-making chain. We recommend prioritizing deployment of the LightGBM model in inpatient systems to achieve automated, high-risk screening for large-scale patient data. When the system identifies high-risk individuals, it will not only output an early warning signal but also generate a personalized risk factor analysis report, clearly labeling the main risk sources and their contributions. It is recommended that nurses develop dynamic assessment protocols for patients based on different risk levels (30) using a stratified application strategy. In clinical environments where interpretability is of primary importance, such as outpatient consultations or shared decision-making with patients, LR remains a viable alternative owing to its straightforward interpretation as odds ratios, which are readily understood by clinicians (Table 8).

**Table 8:** Validation AUC comparison of four models with two missing value imputation methods.

Model	MICE_AUC	Complete_Case_AUC	AUC_Diff
LR	0.854	0.843	−0.011
LightGBM	0.862	0.858	−0.004
RF	0.853	0.850	−0.003
XGBoost	0.848	0.844	−0.004

Nevertheless, before clinical deployment of either model, further prospective multicenter validation with adequately powered sample sizes is essential to confirm the generalizability of our findings. Future research should also focus on evaluating the real-world clinical impact of model-guided antiemetic prophylaxis through prospective utility studies and cluster-randomized trials.

### Limitations

Several limitations of this study should be acknowledged. First, the retrospective single-center design may introduce selection bias and limit generalizability to other institutions, patient populations, and perioperative protocols. Second, while we used LASSO regularization to mitigate overfitting, the limited sample size may affect model stability and the precision of performance estimates. Third, the relatively small validation cohort further limits the precision of performance estimates and may affect the stability of model comparisons. Fourth, the validation cohort originated from the same institution, involving similar surgical teams, perioperative protocols, and patient populations; therefore, this constitutes internal temporal validation rather than true external validation. Fifth, we were unable to compare our model with established clinical scores, due to limitations in retrospective data collection. Future studies with larger, multicenter cohorts and prospective designs are needed to address these limitations and further validate our findings.

## Conclusion

PONV risk prediction models were constructed utilizing multiple feature variables from collected clinical data based on four ML algorithms: LR, RF, XGBoost, and LightGBM. Among these, the LightGBM model demonstrated superior predictive performance. This approach facilitates the early identification of patients at potential risk for PONV and enables the formulation of scientifically sound, targeted interventions based on significant risk factors, thereby aiming to reduce the incidence of PONV, enhance the quality of life for patients following craniotomy, and improve patient satisfaction.

Nevertheless, this study has certain limitations. As a retrospective, single-center study, selection bias may exist, and the temporal generalization ability of the model must be verified. Future multi-center, large-sample prospective studies are recommended to further optimize the model structure and enhance the validation efficiency. Meanwhile, health economic evaluations should be supplemented to quantify the economic benefits of the model to reduce over-treatment, lower the missed diagnosis rate, and optimize the allocation of medical resources, thereby providing solid economic support for extensive clinical transformation of the model.

## Data Availability

The raw data supporting the conclusions of this article will be made available by the authors, without undue reservation.
